# A four‐gene‐based prognostic model predicts overall survival in patients with hepatocellular carcinoma

**DOI:** 10.1111/jcmm.13863

**Published:** 2018-09-24

**Authors:** Junyu Long, Lei Zhang, Xueshuai Wan, Jianzhen Lin, Yi Bai, Weiyu Xu, Jianping Xiong, Haitao Zhao

**Affiliations:** ^1^ Department of Liver Surgery Peking Union Medical College Hospital Chinese Academy of Medical Sciences and Peking Union Medical College Beijing China

**Keywords:** hepatocellular carcinoma, overall survival, risk score

## Abstract

With the development of new advances in hepatocellular carcinoma (HCC) management and noninvasive radiological techniques, high‐risk patient groups such as those with hepatitis virus are closely monitored. HCC is increasingly diagnosed early, and treatment may be successful. In spite of this progress, most patients who undergo a hepatectomy will eventually relapse, and the outcomes of HCC patients remain unsatisfactory. In our study, we aimed to identify potential gene biomarkers based on RNA sequencing data to predict and improve HCC patient survival. The gene expression data and clinical information were acquired from The Cancer Genome Atlas (TCGA) database. A total of 339 differentially expressed genes (DEGs) were obtained between the HCC (n = 374) and normal tissues (n = 50). Four genes (CENPA, SPP1, MAGEB6 and HOXD9) were screened by univariate, Lasso and multivariate Cox regression analyses to develop the prognostic model. Further analysis revealed the independent prognostic capacity of the prognostic model in relation to other clinical characteristics. The receiver operating characteristic (ROC) curve analysis confirmed the good performance of the prognostic model. Then, the prognostic model and the expression levels of the four genes were validated using the Gene Expression Omnibus (GEO) dataset. A nomogram comprising the prognostic model to predict the overall survival was established, and internal validation in the TCGA cohort was performed. The predictive model and the nomogram will enable patients with HCC to be more accurately managed in trials testing new drugs and in clinical practice.

## 1 INTRODUCTION

Hepatocellular carcinoma (HCC) is one of the most frequently diagnosed malignancies and the third leading cause of cancer‐related mortality, and the incidence and mortality of this cancer are increasing yearly.^1^ Importantly, the 5‐year survival rate of patients with HCC is only 12%. Therefore, prognostic biomarkers are needed to help predict the outcome for HCC patients and to outline an individualized treatment plan. Age, clinical staging and vascular tumour invasion are important contributors to clinical outcomes and may also help improve survival forecasts.^2^ However, traditional clinical information has a limited predictive power because of the complex molecular mechanisms of tumour regulation. Therefore, there is a strong need to explore new tools, such as molecular markers, to predict patient prognosis more accurately.

In this genomic era, a large number of genome‐sequencing technologies and data have emerged.^3^ These tools have made great contributions to tumour diagnosis and prognosis prediction. RNA sequencing is a recently developed deep‐sequencing method that can recognize splice variants, unmapped genes and spliced unrecognized noncoding RNAs. Using the lung adenocarcinoma (LUAD) dataset of The Cancer Genome Atlas (TCGA), Anlin Feng et al^4^ found that the overall survival (OS) of LUAD patients with high HMGB1 expression was poor, demonstrating that these novel next‐generation sequencing approaches and data can identify clinical biomarkers of cancer. Similarly, we predicted the prognosis of HCC patients using high‐throughput sequencing of biomarkers.

In this study, we aimed to explore the difference in the mRNA expression profiles of HCC and the adjacent liver to identify potential gene biomarkers using TCGA data. We established a four‐gene prognostic model that included CENPA, SPP1, MAGEB6 and HOXD9 based on our RNA sequencing survival analysis. The risk‐coefficient model was validated in the Gene Expression Omnibus (GEO). Functional analysis of the predictive genes was performed. The expression profiles of the predictive genes were verified in GEO and by immunohistochemical staining with HCC samples in the Human Protein Atlas. A predictive nomogram was built and internally validated in the TCGA cohort. As a whole, this prognostic model and nomogram might be helpful in guiding the prognostic status of patients with HCC.

## 2 MATERIALS AND METHODS

### 2.1 Data source

The mRNA expression profiles and the corresponding clinical information from the patients with HCC were obtained from the TCGA, which was calculated on an Illumina HiSeq RNA‐seq platform, containing 374 HCC tissues and 50 adjacent nontumourous liver tissues as of January 1, 2018. The data from the TCGA are publicly available and open‐access; therefore, the local ethics committees did not need to approve the study because the current research follows the TCGA data access policies and publication guidelines.

### 2.2 Differentially expressed mRNA screening between HCC and noncancer tissues

First, we obtained the raw counts of the HCC mRNA expression profiles from the TCGA databases. The RNA sequencing data of HCC included 57 000 mRNA expression profiles. Then, the differentially expressed genes (DEGs) were calculated using the DESeq R package. The DEGs of the dataset with an absolute log2 fold change (FC) > 4 and an adjusted *P* value of <0.001 were considered for subsequent analysis.

### 2.3 Definition of the gene‐related prognostic model

Univariate, Lasso and multivariate Cox regression analyses were employed to investigate the correlation between patient OS and the expression level of each gene. The gene was considered significant when the *P* value was <0.001 in the univariate Cox regression analysis. Next, we applied a Lasso‐penalized Cox regression to further reduce genes for patients with HCC. For the Lasso‐penalized Cox regression selection operator, we subsampled the dataset with replacement 1000 times and selected the markers with repeat occurrence frequencies of more than 900. The tuning parameters were determined according to the expected generalization error estimated from 10‐fold cross‐validation and information‐based criteria Akaike Information Criterion/Bayesian Information Criterion (AIC/BIC), and we adopted the largest value of lambda such that the error was within one standard error of the minimum, called “1‐se” lambda. Finally, a multivariate Cox regression analysis was conducted to assess the contribution of a gene as an independent prognostic factor for patient survival. A stepwise method was employed to further select the best model. A four‐gene‐based prognosis risk score was established based on a linear combination of the regression coefficient derived from the multivariate Cox regression model (β) multiplied with its expression level. The Prognosis Index (PI) = (β* expression level of CENPA) + (β* expression level of SPP1) + (β* expression level of MAGEB6) + (β* expression level of HOXD9). The optimal cut‐off value was found using X‐tile software.^5^ Thresholds for the scores that were outputted from the prognostic model that were applied to classify patients into low‐ and high‐risk groups were defined as the scores that yielded the largest *χ*² value in the Mantel‐Cox test. The 365 HCC patients with survival data were separated into low‐ and high‐risk groups based on the optimal cut‐off value. The Kaplan‐Meier (K‐M) survival curves for the cases with a low or high risk were produced. Time‐dependent receiver operating characteristic (ROC) curve analyses were conducted to evaluate the predictive power of the prediction model. Then, the prognostic model was validated in the GEO dataset (GSE54236).

### 2.4 Independence of the prognostic model from other clinical characteristics

To determine whether the predictive power of the prognostic model could be independent of other clinical variables (including age, AFP, sex, weight, inflammation, histologic grade, family history, pathologic stage, tumour grade and vascular tumour invasion) for patients with HCC, univariate and multivariate Cox regression analyses were conducted, with the other traditional clinical characteristics as independent variables and the OS as the dependent variable. All reported *P* values were two‐sided. The hazard ratio (HR) and 95% confidence intervals were calculated.

### 2.5 Functional enrichment analysis

The Gene Ontology (GO) and Kyoto Encyclopedia of Genes and Genomes (KEGG) pathway enrichment analyses of the 4 protein‐coding genes were performed using the Database for Annotation, Visualization and Integrated Discovery (DAVID) Bioinformatics Tool, version 6.8.

### 2.6 External validation of the expression patterns of the four genes

We also attempted to validate the expression patterns of the four genes in the TCGA; thus, the expression levels of these four mRNAs from GEO (GSE54236) were extracted for further analysis. The differentially expressed patterns of the four genes between the HCC and nontumourous tissues were analysed in Prism 6.0 (GraphPad, San Diego, CA, USA) using the Wilcoxon signed‐rank test. The *P* values are two‐sided, and *P* < 0.05 indicates statistical significance.

### 2.7 Building and validating a predictive nomogram

Nomograms are widely applied to predict cancer patients’ prognoses, mainly because they can reduce the statistical prediction models into a single numerical assessment of the probability of OS that is tailored to the profile of an individual patient.^6^ In this study, the combined model based on all independent prognostic factors selected by the multivariable Cox regression analysis was used to construct a nomogram to assess the probability of 1‐, 3‐, and 5‐year OS for patients with HCC. Subsequently, validations, including discrimination and calibration, were performed. The discrimination of the nomogram was calculated using the concordance index (C‐index) by a bootstrap method with 1000 resamples. The value of the C‐index ranged from 0.5 to 1.0, of which 1.0 indicates a perfect capacity to correctly distinguish the outcome with the model and 0.5 indicates a random chance. The calibration curve of the nomogram was evaluated graphically by plotting the nomogram prediction probabilities against the observed rates. Overlapping with the reference line demonstrated that the model was in perfect agreement. At the same time, we also constructed nomograms built with a single significant prognostic factor and compared the predictive accuracy between them and the combined model using the C‐index, ROC analysis and the decision curve analysis (DCA). DCA was initially used as a novel analytical technique that incorporated the clinical consequences of a decision to quantify the clinical utility of a prediction nomogram. Therefore, the DCA can decide whether the predictive nomogram is clinically useful or not. The best model is one with a high net benefit as calculated within the favourable probability.

## 3 RESULTS

### 3.1 Differentially expressed mRNAs between HCC and normal tissue

**Figure 1:**
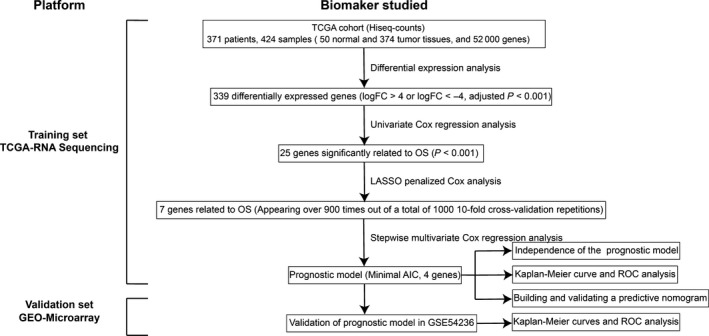
Overall workflow describing the process used to develop and validate the prognostic model to predict prognostic outcomes

To describe our study more clearly, a flow chart of the analysis procedure was developed (Figure 1). A total of 339 differentially expressed mRNAs (logFC > 4 or logFC < −4, adjusted *P* < 0.001) adjusted for false discovery rate were identified in the mRNA expression profiles of HCC tissues (n = 374) compared with normal tissues (n = 50; Figure 1). Among them, 16 mRNAs were overexpressed, and 323 mRNAs were downregulated (Figure S1), which were further investigated with regard to their prognostic value.

### 3.2 The risk stratification and ROC curve indicate a good performance for the prognostic model in predicting the OS of HCC patients

**Figure 2:**
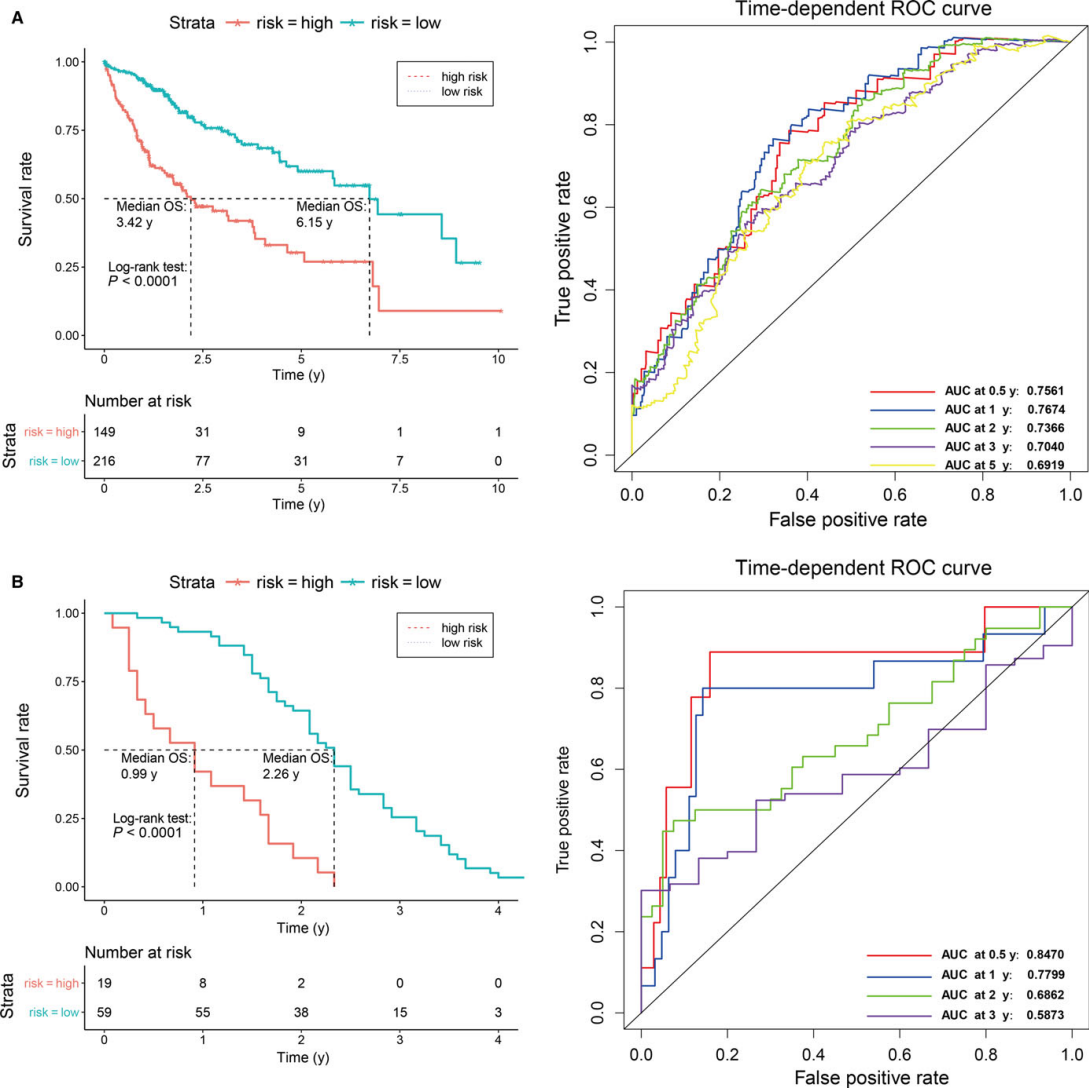
K‐M and time‐dependent ROC curves for the prognostic model in the TCGA HCC cohort (A) and in the GEO HCC cohort. The K‐M survival curves show the overall survival based on the relative high‐ and low‐risk patients divided by the optimal cut‐off point. Time‐dependent ROC curve analysis of survival prediction by the prognostic model

We conducted a univariate Cox regression to investigate the correlation of the differentially expressed genes with the OS of HCC patients and identified 25 genes significantly related to OS in HCC patients when the *P* value was <0.001. Then, after primary filtration, a Lasso‐penalized Cox analysis with penalty parameter tuning performed via 10‐fold cross‐validation was established to further narrow the mRNAs in which we required selected mRNAs to appear over 900 times of a total of 1000 repetitions (Figure S2). As a result, 7 mRNAs were identified. Finally, a stepwise multivariate Cox regression analysis was conducted, and four genes were finally selected to build a predictive model. The predictive model was characterized by the linear combination of the expression levels of the four genes weighted by their relative coefficient in the multivariate Cox regression as follows: risk score = (0.2013 * expression level of CENPA) + (0.0967* expression level of SPP1) + (0.1310 * expression level of MAGEB6) + (0.0937 * expression level of HOXD9). All of the genes, including centromere protein A (CENPA), secreted phosphoprotein 1 (SPP1), MAGE family member B6 (MAGEB6) and homeobox D9 (HOXD9), showed positive coefficients in the Cox regression analysis, implying high‐risk signatures for these four genes, because their high expression signified a shorter OS of the patients. For the 365 patients with survival time in this study, we calculated the four‐gene expression risk score and used X‐tile diagrams to produce the optimal cut‐off value for the risk score (Figure S3 A). A total of 149 patients were classified as high risk because their risk score was greater than the cut‐off value, while the other 216 patients were assigned to the low‐risk group, with risk scores below the cut‐off point (Figure S4 A). The K‐M OS curves of the two groups, based on the four genes, were significantly different (median OS, 3.42 years vs 6.15 years, *P* < 0.0001; Figure 2A). The prognostic capacity of the four‐gene signature was assessed by calculating the AUC of a time‐dependent ROC curve (Figure 2A). The higher the AUC, the better the model performance. The AUCs of the four‐gene biomarker prognostic model were 0.7561, 0. 7674, 0.7366, 0.7040 and 0.6919 for the 0.5, 1, 2, 3 and 5‐year survival times, respectively, indicating that the forecast model had a high sensitivity and specificity (Figure 2A).

### 3.3 The prognostic model is independent of conventional clinical factors

**Figure 3:**
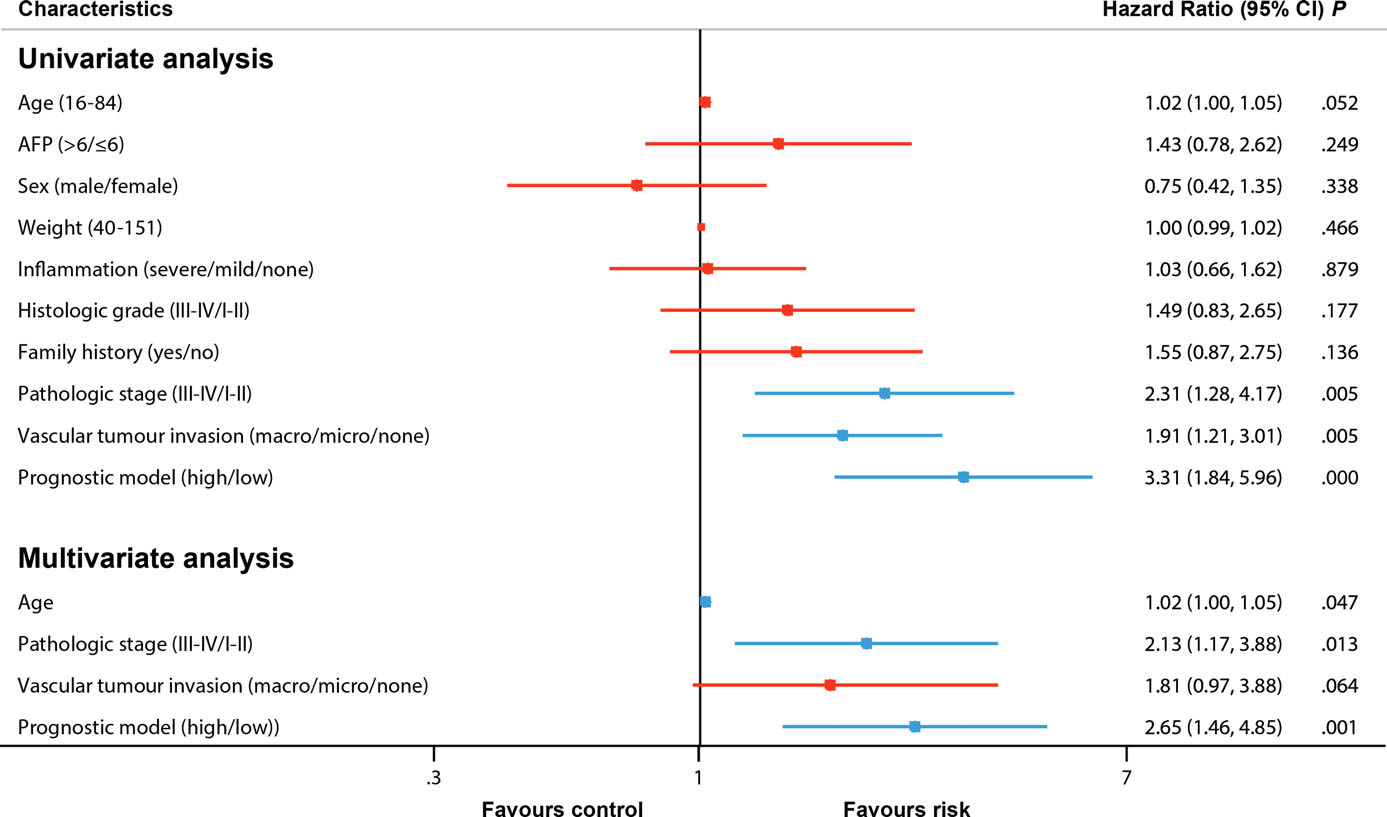
Univariate and multivariate association of the prognostic model and clinicopathological characteristics with overall survival. Red represents no statistical significance, and blue represents statistical significance

Univariate and multivariate Cox regression analyses were used to evaluate the independent predictive value of the four‐gene prognostic model in 173 HCC patients with complete clinical information from the TCGA HCC cohort. The prognostic model and clinical covariates of pathologic stage and vascular tumour invasion had some prognostic value with the univariate Cox regression analysis. However, age, AFP, sex, weight, inflammation, histologic grade and family history did not correlate with OS (Figure 3; Table S1). Considering that age almost reached statistical significance, we incorporated age, pathologic stage, vascular tumour invasion and the prognostic model into the multivariate Cox regression analysis. After the multivariate Cox regression analysis, the age, the pathologic stage and the prognostic model were independent prognostic factors associated with OS (Figure 3; Table S1).

### 3.4 Prediction condition of the prognostic model in the GEO dataset

To evaluate the predictive value of the prognostic model in predicting the OS for the patients with HCC in other datasets, the prognostic model was assessed in the GEO microarray data (GSE54236).^7^ A total of 78 patients in the GSE54236 data were classified into a low‐risk group (n = 59) and a high‐risk group (n = 19) using the optimal risk cut‐off value of the GEO data and the same risk score model of the TCGA data (Figures S3B and S4B). Consistent with the result in the TCGA, the OS of the HCC patients in the GSE54236 data in the high‐risk group was significantly lower than that in the low‐risk group (median survival: 0.99 years vs 2.26 years; *P* < 0.0001; Figure 2B). The 3‐year survival rates were 25% in low‐risk patients and 0% in high‐risk patients. In addition, the time‐dependent ROC analyses for the survival prediction of the prognostic model obtained AUCs of 0.8470 at 0.5 years, 0.7799 at 1 year, 0.6862 at 2 years and 0.5873 at 3 years, demonstrating that this prognostic model was capable of predicting OS in HCC patients (Figure 3B). The AUC at 5 years was not shown in Figure 2B because no patients in the GEO validation set survived for more than 5 years.

### 3.5 Functional enrichment analysis

To elucidate the functional characteristics of the identified protein‐coding genes, we performed enrichment analyses of the GO and KEGG pathways, which showed that a total of 1 GO term and 4 KEGG pathways were enriched by the four‐gene signature (*P* < 0.05; Figure S5). The results showed that the genes were enriched in skeletal system development function and ECM‐receptor interaction, the Toll‐like receptor signalling pathway, focal adhesion and the PI3K‐Akt signalling pathway.

### 3.6 Validation of the expression of the four mRNAs

**Figure 4:**
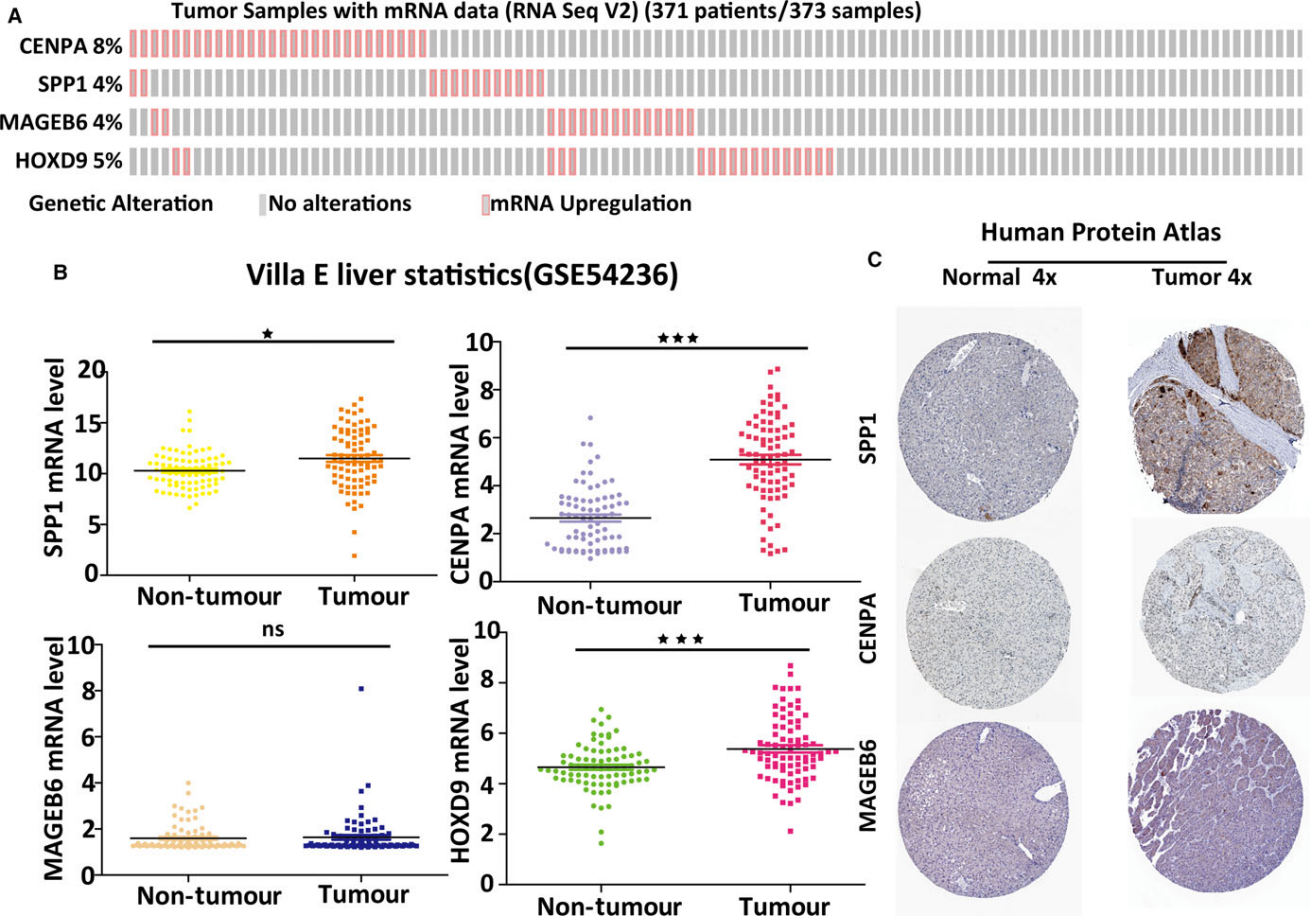
The four prognostic genes are upregulated in human HCC specimens. A, The expression profiles of the four genes in the TCGA liver cancer RNA‐seq (n = 371) dataset. B, GEO data showing the expression profiles of the four prognostic genes in normal liver (n = 78) vs tumour tissue (n = 78); *P* < 0.01 (*), *P* < 0.001 (**) and *P* < 0.0001 (***). C, The expression profiles of the four genes in the normal liver tissue and HCC specimens. Images were taken from the Human Protein Atlas (http://www.proteinatlas.org) online database

In the TCGA HCC cohort, all four genes were highly expressed in HCC compared with their expression in the adjacent nontumourous liver tissues (Figure 4A). To further confirm the expression patterns of the four genes in the GEO database, they were selected from GSE54236. The transcriptome profiling data in the GSE54236 dataset contained 78 patients, including 78 adjacent nontumour samples and 78 HCC samples. Consistent with our results, the mean expression levels of CENPA, SPP1 and HOXD9 were significantly higher than those of noncancerous liver tissues in the GEO database (Figure 4B). However, due to the small size of the patients, no significant differences were found in MAGEB6 expression (Figure 4B). To determine the clinical relevance of the expression of the four genes, we analysed the expression of the proteins encoded by the four genes using clinical specimens from the Human Protein Profiles (http://www.proteinatlas.org).^8^ SPP1 was strongly positive in HCC and CENPA, and MAGEB6 was weakly positive in HCC, relative to their expression levels in normal liver tissue (Figure 4C). However, HOXD9 was not found on the website.

### 3.7 Building and validating a predictive nomogram

**Figure 5:**
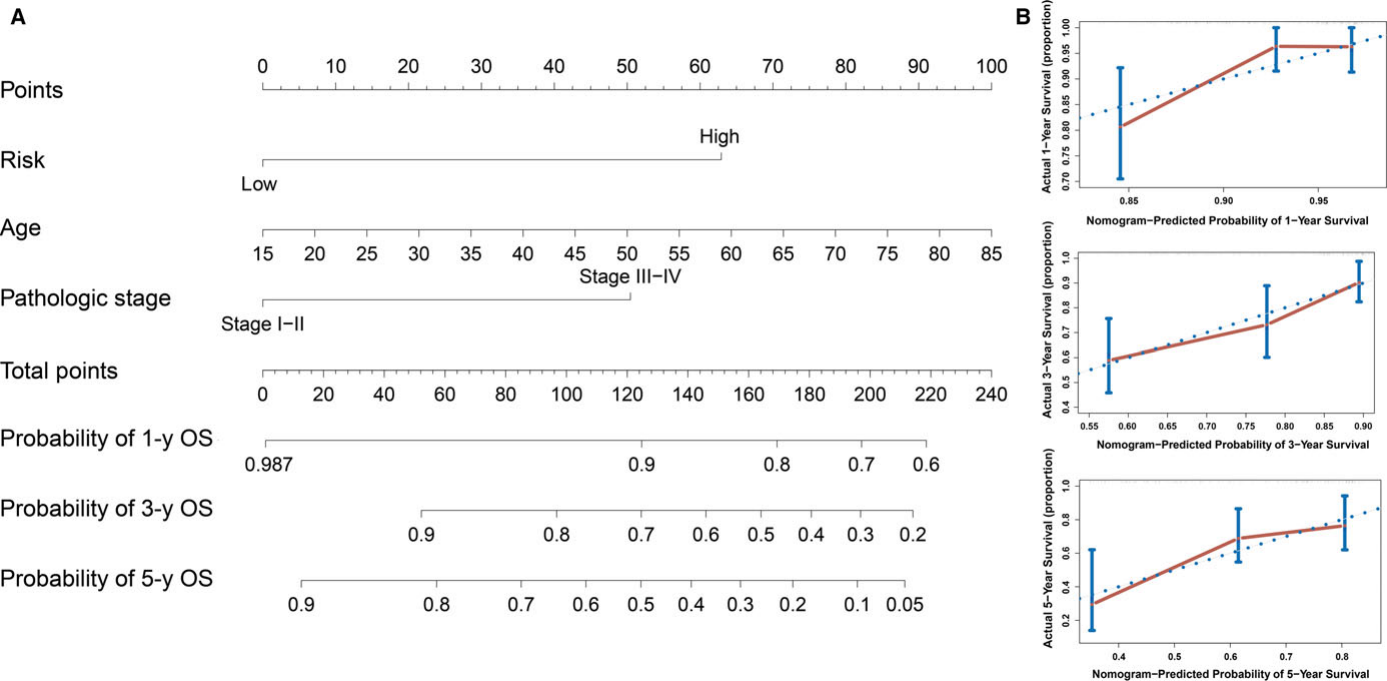
Nomogram predicting 1‐, 3‐ and 5‐y OS for patients with HCC (A). The nomogram is applied by adding up the points identified on the points scale for each variable. The total points projected on the bottom scales indicate the probability of 1‐, 3‐ and 5‐y OS. The calibration curve for predicting 1‐, 3‐ and 5‐y OS for patients with HCC (B). The *Y*‐axis represents actual survival, as measured by K‐M analysis, and the *X*‐axis represents the nomogram‐predicted survival

**Figure 6:**
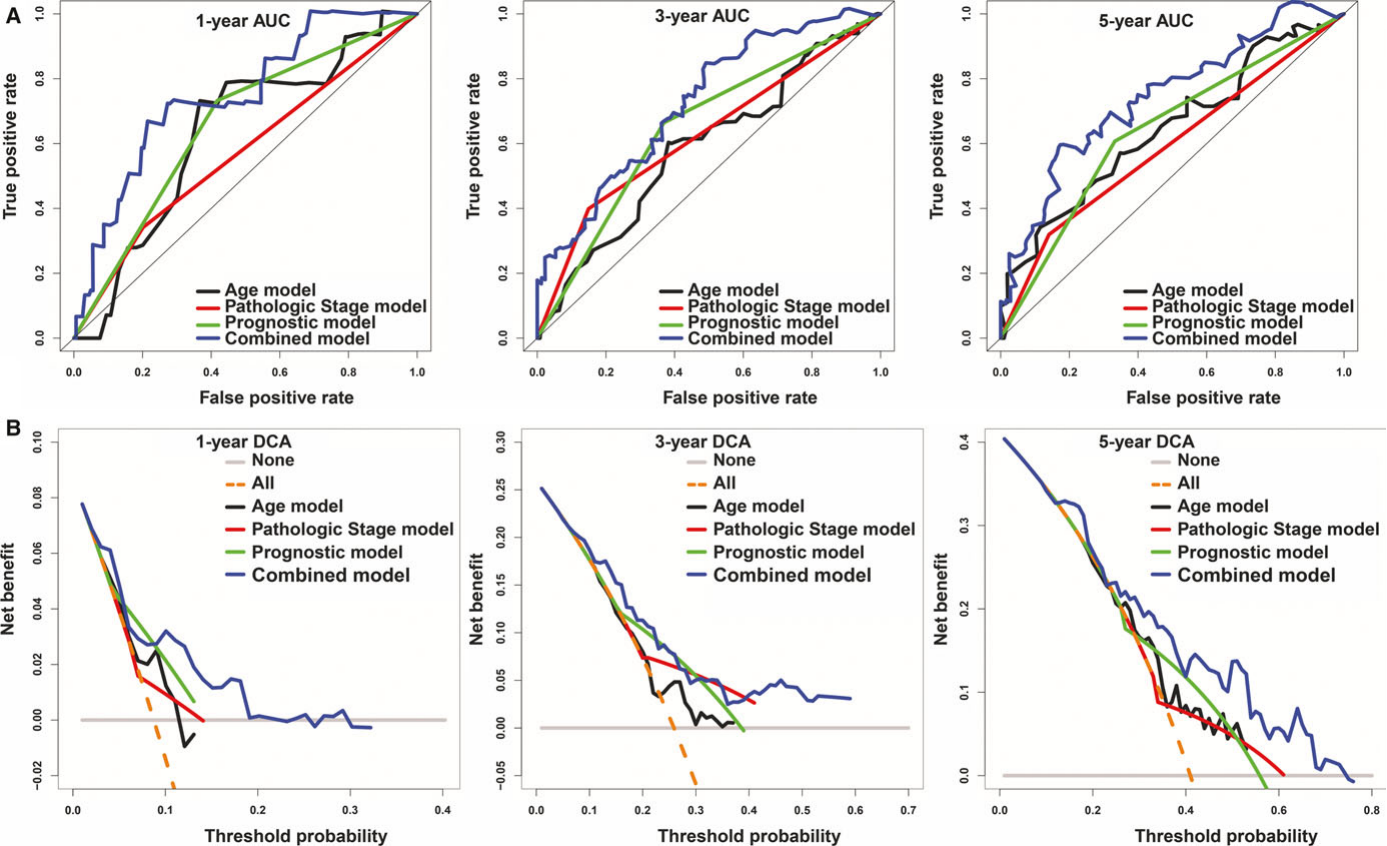
The time‐dependent ROC and DCA curves of the nomograms. Time‐dependent ROC curves analysis evaluates the accuracy of the nomograms (A). The black, red, green or blue solid line represents the nomogram. The DCA curves can intuitively evaluate the clinical benefit of the nomograms and the scope of application of the nomograms to obtain clinical benefits (B). The net benefits (*Y*‐axis) as calculated are plotted against the threshold probabilities of patients having 1‐, 3‐ and 5‐y survival on the *X*‐axis. The orange dotted line represents the assumption that all patients have 1‐, 3‐ and 5‐y survival. The grey solid line represents the assumption that no patients have 1‐, 3‐ or 5‐y survival. The black, red, green or blue solid lines represent the nomograms

To establish a clinically applicable method for predicting the survival probability of patients with HCC, we developed a nomogram to predict the probability of the 1‐, 3‐ and 5‐year OS in the TCGA cohort. The predictors of the nomogram included three independent prognostic factors (age, pathologic stage and prognostic model; Figure 5A). The C‐index for the model for evaluation of OS was 0.69, with 1000 cycles of bootstrapping (95% confidence interval: 0.61, 0.77). Calibration plots were used to visualize the performances of the nomograms. The 45° line represented the best prediction. Calibration plots showed that the nomogram performed well (Figure 5B). At the same time, we compared the accuracy of the prediction between this nomogram and the age, pathologic stage and prognostic model. Compared with the age, pathologic stage and prognostic model, the performance of nomogram discrimination was significantly higher than that of the age, pathologic stage and prognostic model (Table 1). The AUC of the nomogram was also the largest (Figure 6A). The clinical usefulness was assessed using DCA. The nomogram showed the best net benefit (Figure 6B). These findings demonstrate that compared with nomograms built with a single prognostic factor, the nomogram built with the combined model is the best nomogram for predicting survival for patients with HCC, whether in the short or long term, which might facilitate patient counselling, decision‐making and follow‐up scheduling.

**Table 1:** Comparison of the nomogram with age, pathologic stage and the combined model

Models	C‐index (95%, CI)	*P*‐value
Age model	0.58 (0.49‐0.67)	‐
Pathologic stage model	0.57 (0.50‐0.64)	‐
Prognostic model	0.65 (0.57‐0.72)	‐
Nomogram (Combined model)	0.69 (0.61‐0.77)	‐
Nomogram vs age model	‐	<0.05
Nomogram vs pathologic stage model	‐	<0.01
Nomogram vs prognostic model	‐	<0.05

## 4 DISCUSSION

HCC remains one of the deadliest malignant tumours worldwide because of its complicated molecular and cellular heterogeneity, and its incidence increases every year.^9^ Therefore, understanding HCC biology may offer clinicians new tools that can be used to treat the disease. Comprehensive genomic studies showing the effects of RNA have received considerable attention. Many potential and valuable mRNAs must be identified to improve the clinical outcome for HCC patients. However, the number of specific biomarkers that can be used to show therapeutic effects is still small, and prognostic factors are important for the treatment of HCC patients. Therefore, to reduce mortality and improve HCC prognosis, there is an urgent need for the molecular screening of biomarkers of HCC.

In our study, we investigated the differences in gene expression between HCC and adjacent nontumour liver tissues to identify potential gene biomarkers using the TCGA database. The differentially expressed genes were screened, and univariate, Lasso and multivariate Cox analyses were conducted to build a risk model to predict HCC prognosis. We identified four genes: CENPA, SPP1, MAGEB6 and HOXD9. High expression levels of CENPA, SPP1, MAGEB6 and HOXD9 were relevant to a poor prognosis in HCC patients. The AUCs of the ROC curve for the prognostic model for predicting the 0.5, 1, 2, 3 and 5‐year survival were 0.7561, 0. 7674, 0.7366, 0.7040 and 0.6919, respectively, indicating that the four‐gene signature had a good performance for survival prediction. With the mRNA‐based risk scoring prognostic model, the patients with HCC were divided into a high‐risk group and a low‐risk group. The clinician can change the patient's treatment plan based on the predicted outcome of the model to achieve individualized treatment of liver cancer patients. Strategies should be established to prevent or detect HCC recurrence early in high‐risk populations. Therefore, high‐risk groups should be followed more frequently, and chest and abdomen CT scans should be performed regularly to diagnose the recurrence of HCC early. The prognostic model may also be a useful guide to determine the organ allocation for secondary liver transplantation of HCC patients initially treated with partial hepatectomy. Patients with poor prognosis predicted by the prognostic model may not be suitable for cadaveric liver transplantation.

We also demonstrated that the prognostic model was independent of other clinical factors in HCC. The prognostic model was used to predict the GEO dataset (GSE54236) to test its predictive power, and the expression levels of the four genes were also validated. Using the enrichment and functional analysis and the DAVID and KEGG bioinformatics tools, we found that the GO functions of the four genes were enriched in skeletal system development (*P* value <0.05), and the KEGG pathways were enriched in ECM‐receptor interaction, the Toll‐like receptor signalling pathway, focal adhesion and the PI3K‐Akt signalling pathway.

A nomogram is a statistical tool that provides the individual patient with the overall probability of a particular outcome. In this study, we constructed a nomogram built with a combined model to accurately predict the likelihood of OS in patients with HCC. The calibration plots indicated that actual survival corresponded closely with predicted survival, suggesting that the predictive performance of the nomogram was good. Meanwhile, we demonstrated that the nomogram built with the combined model is the best by C‐index, AUC and DCA compared with other nomograms built with a single risk factor.

SPP1 (also known as osteopontin) is a multifunctional cytokine expressed by cells from various tissues.^10^ SPP1 takes part in many physiological and pathological processes, including drug resistance, cell proliferation, invasion, survival, stem‐like behaviour and tumour metastasis. SPP1 overexpression effectively increases HCC growth and metastasis.^11^

The HOX gene family is a network of genes that encode DNA‐binding proteins.^12^ This family is highly conserved throughout the whole evolution process and is involved in many signal transduction pathways, such as cell development, migration and differentiation.^12^, ^13^ HOXD9 is a member of the homeobox gene family.^14^ HOXD9 showed high upregulation in invasive HCC cells. The high expression of HOXD9 is related to the invasion, migration and metastasis of HCC. HOXD9 leads to mesenchymal‐epithelial transition (MET), and silencing HOXD9 promotes MET in liver cancer cells. HOXD9 may be an effective therapeutic target for HCC.

MAGEB6, a protein‐coding gene, is expressed in testis tissue and a wide variety of different histological types of tumours.^15^ MAGEB6 belongs to the MAGEB gene family.^15^ For all members of this gene family, the entire coding sequence is located in the last exon, and the sequence identity of encoded proteins reaches 50%‐68%.^16^, ^17^ Cancer‐testis antigens (CTAs) are expressed in diverse histological types of malignant tumours, yet they are seldom expressed in normal somatic tissues except for immunoprivileged gametogenic tissues, which indicates a similarity between the gametogenesis and tumourigenesis processes.^18^ CTAs exhibit a strong immunogenicity and a specific expression pattern, making them a promising target.^19^ CTAs or peptides derived from CTAs can be used for cancer vaccination due to their immunogenicity.^19^ Currently, there are over 100 identified CT genes from 44 different families.^20^ Due to the special expression pattern and antigenicity of CT genes, an increasing number of CTA‐related biomarkers and therapeutic cancer vaccines have been reported for use in early clinical diagnosis as well as prognostic prediction.^21^, ^22^ Peptides derived from CTAs are being extensively tested in clinical trials for many types of cancers, such as lung cancer and head and neck cancer.^23^, ^24^ In HCC, a large number of CTA genes show validated expression at the RNA level, including members of the MAGE‐A, MAGE‐B, and MAGE‐C families.^25^ As of now, the best‐characterized CTAs in HCC are the MAGE antigens.^26^ In particular, up to 45%‐52% of all HCCs express the MAGEB1 antigen, and up to 60%‐62% of all HCCs express the MAGEB2 antigen.^26^ However, the expression pattern of MAGEB6 in HCC has not previously been elucidated. In this study, we show for the first time that MAGEB6 is highly expressed in HCC based on RNA‐seq data in the TCGA database, and its use in the prognostic model was found to independently predict the prognosis of patients with HCC, similar to its predictive utility in head and neck squamous cell carcinoma (HNSCC). MAGEB6 was found to be frequently expressed in HNSCC, and there is a clear association between MAGE6B mRNA positivity and recognized clinical characteristics of unfavourable prognosis, which suggests that MAGE6B may be an interesting target for HNSCC immunotherapy treatment.^27^ Therefore, because CTAs are capable of eliciting spontaneous antitumour immune responses, the CTA member MAGEB6 is a promising candidate biomarker for future HCC immunotherapy and is of great importance in the development of cancer vaccines for clinical trials.

To our knowledge, the four‐gene biomarkers have not been previously studied and will provide some clinical indications for the development of new prognostic factors for HCC. One of the advantages of our predictive genes is that they do not require the identification of somatic mutations in patients. In addition, our approach greatly reduces the cost of sequencing, which makes the application of targeted sequencing based on specific genes more cost‐effective and routine. In future, we plan to use single cell transcriptome sequencing in circulating tumour cells to detect the expression of these four genes in patients who are poor candidates for surgery. Accurate prognostic evaluation is crucial for selecting the suitable treatment. In routine clinical practice, pathologic stage is a key prognostic determinant in HCC for oncologists and patients.^28^ Nevertheless, clinical outcomes differ among patients with the same cancer stages, indicating that current staging systems are insufficient for prognosis. The current pathologic stage is entirely based on the anatomical extent of the disease, and the staging system cannot fully reflect the biological heterogeneity of HCC patients. These problems may influence the prediction accuracy of traditional systems for HCC patients. Our nomogram is the first model to combine genetic information with HCC clinical data to predict patient outcomes. Compared with the traditional pathologic stage, the predictive power of the nomogram is increased by 12% (C‐index: 0.57 vs 0.69; *P *< 0.01), and this nomogram may become routinely used in the future.

However, several limitations of the current study should be considered. First, the population ethnicities in the TCGA database are mainly confined to White people and Black people, and extrapolating the findings to other ethnic groups needs to be substantiated. Second, our nomogram did not perform external validation in the GEO database because the GSE54236 lacks clinical data, and a robust nomogram should be validated externally in different cohorts; thus, the nomogram needs to be further validated in multicenter clinical trials and prospective studies. In future, we will also explore the possibility of containing more prognostic variables to further improve performance. Other regression modelling methods will be applied to determine whether predictive accuracy can be further improved.

To sum up, our research results indicate that the four‐mRNA prognostic model is a reliable tool for predicting the OS of HCC patients, and a nomogram comprising a prognostic model can assist clinicians in selecting personalized treatment for patients with HCC.

## CONFLICT OF INTEREST

The authors declare no conflicts of interest.

## AUTHOR CONTRIBUTIONS

JL, LZ and XW analysed data and wrote the manuscript. JL, YB, WX and JX helped to prepare the dataset and participated in the discussion. HZ designed the study. All authors read and approved the final manuscript.
