# Supplementary figures and images for: A four‐gene‐based prognostic model predicts overall survival in patients with hepatocellular carcinoma
Source: J Cell Mol Med. 2018 Sep 24;22(12):5928–38. doi: 10.1111/jcmm.13863 (PMC6237588; doi:10.1111/jcmm.13863)

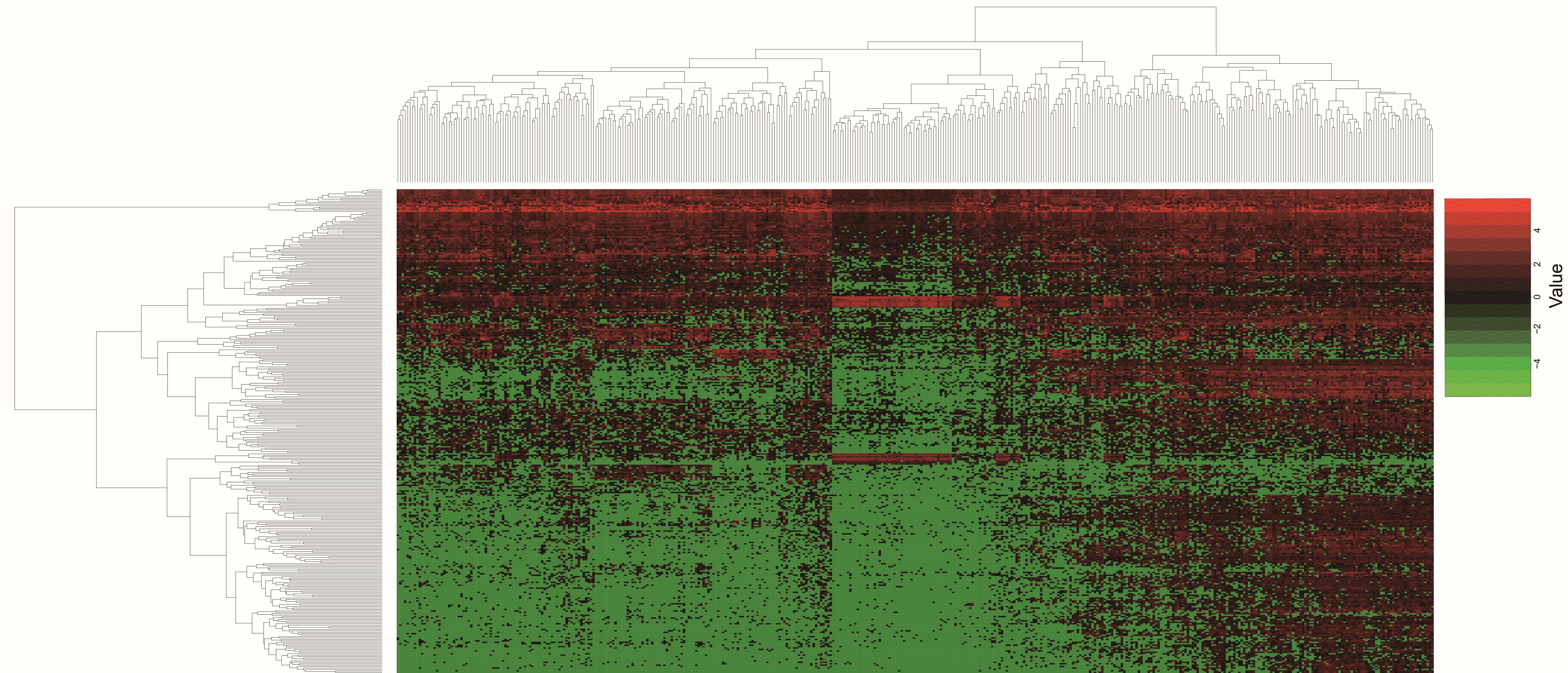

Supplement: Supplementary file 1 [file JCMM-22-5928-s001.tif]

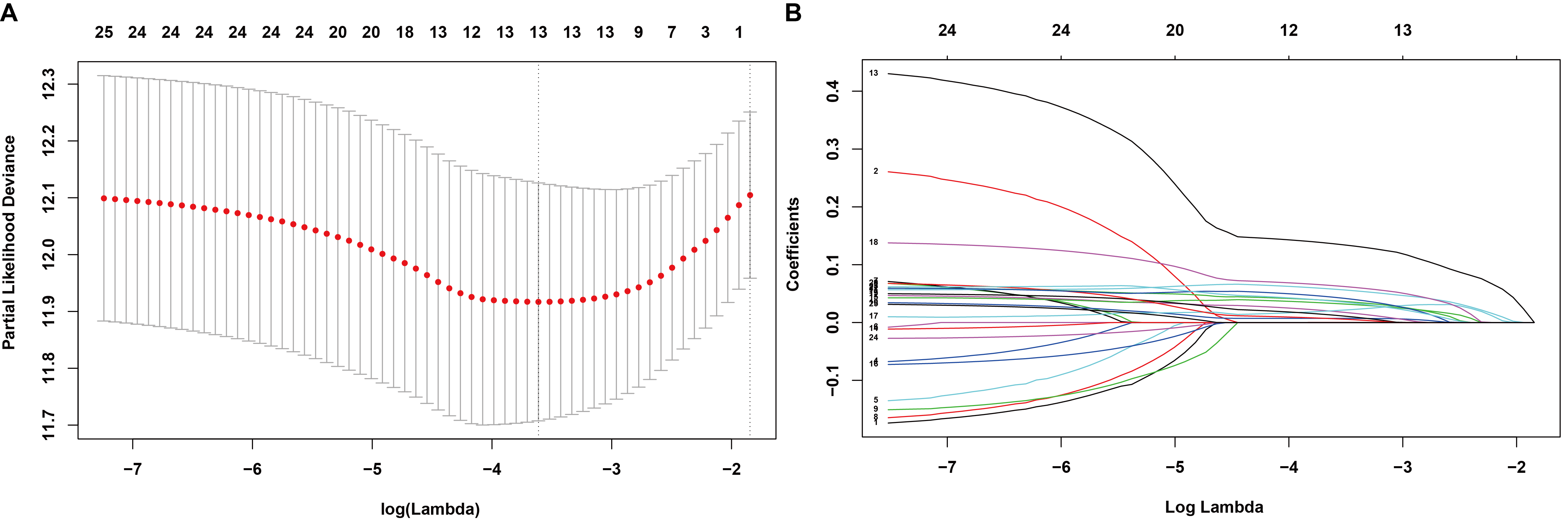

Supplement: Supplementary file 2 [file JCMM-22-5928-s002.tif]

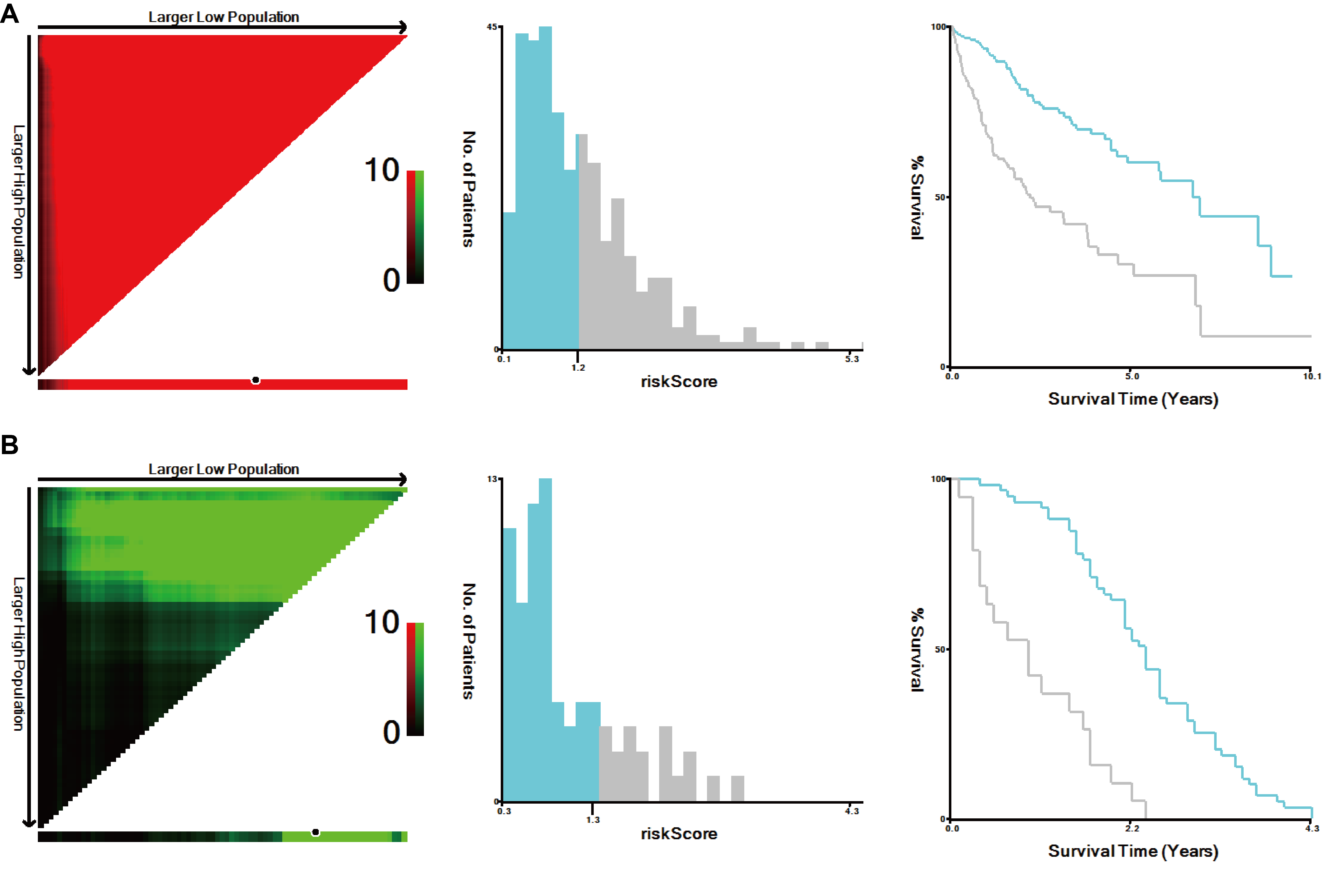

Supplement: Supplementary file 3 [file JCMM-22-5928-s003.tif]

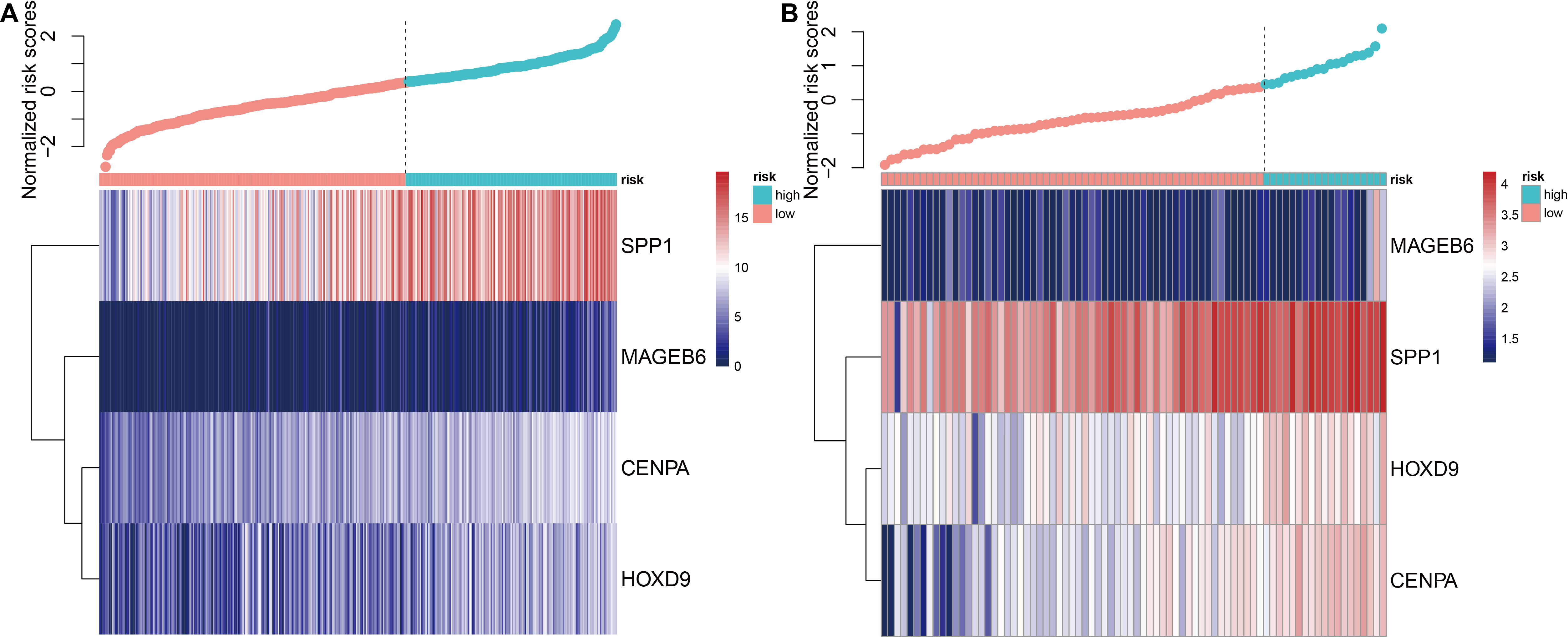

Supplement: Supplementary file 4 [file JCMM-22-5928-s004.tif]

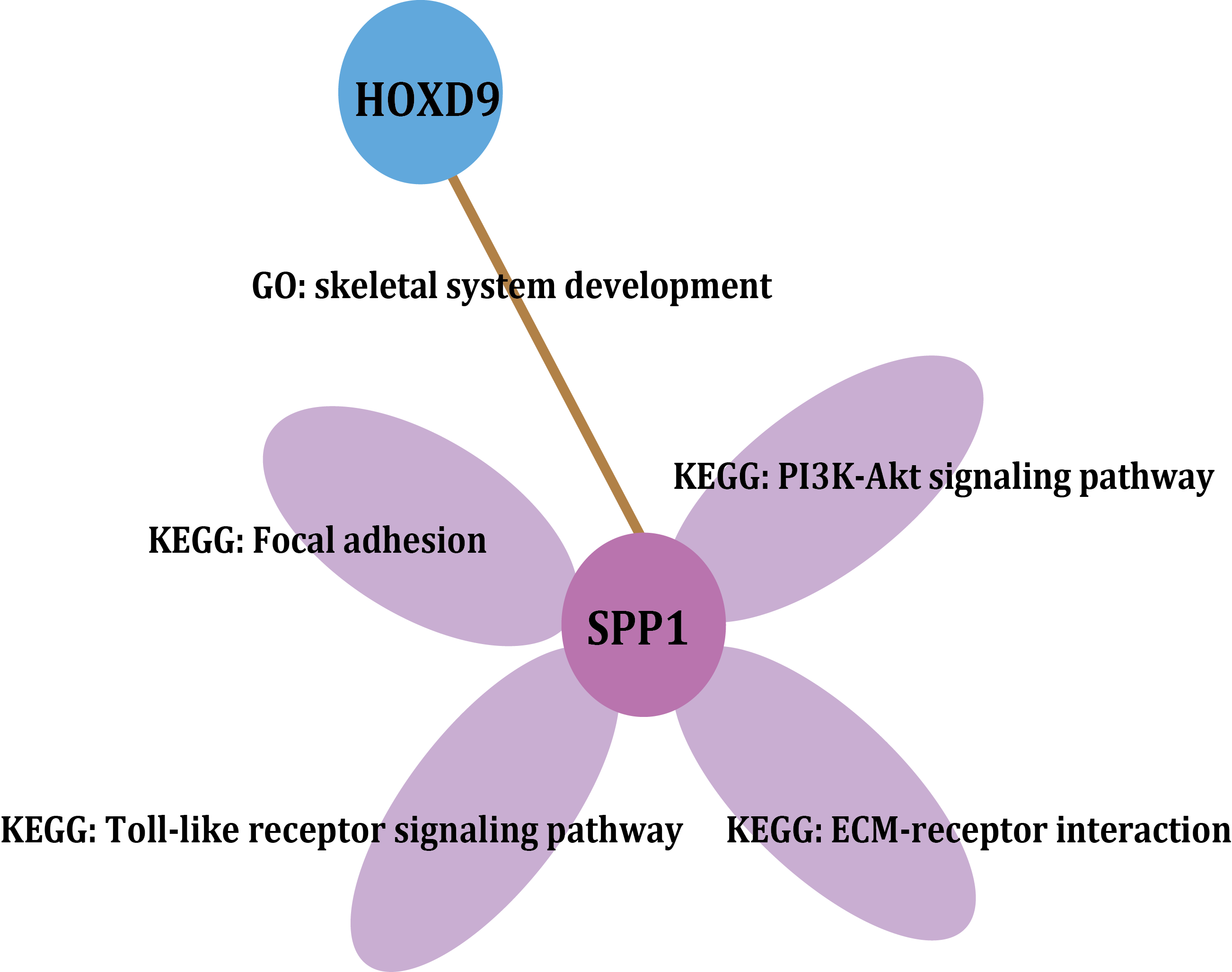

Supplement: Supplementary file 5 [file JCMM-22-5928-s005.tif]
