# Supplementary material for: A four‐gene‐based prognostic model predicts overall survival in patients with hepatocellular carcinoma
Source: J Cell Mol Med. 2018 Sep 24;22(12):5928–38. doi: 10.1111/jcmm.13863 (PMC6237588; doi:10.1111/jcmm.13863)
Supplement: Supplementary file 6 [file JCMM-22-5928-s006.docx]

Table S1: Univariate and multivariate Cox regression analyses of the characteristics and the prognostic model in HCC.

| Characteristics | Univariate analysis | | Multivariate analysis | |
| --- | --- | --- | --- | --- |
|  | HR (95%CI) | P-value | HR (95%CI) | P-value |
| Age (16~84) | 1.023(1.000-1.048) | 0.052 | 1.024 (1.000-1.048) | 0.047 |
| AFP (>6/≤6) | 1.428(0.779-2.617) | 0.249 |  |  |
| Sex (male/female) | 0.750(0.416-1.352) | 0.338 |  |  |
| Weight (40~151) | 1.005(0.991-1.020) | 0.466 |  |  |
| Inflammation (severe/mild/none) | 1.035(0.662-1.619) | 0.879 |  |  |
| Histologic grade (III–IV/I–II) | 1.488(0.835-2.653) | 0.177 |  |  |
| Family history (yes/no) | 1.549(0.872-2.751) | 0.136 |  |  |
| Pathologic stage (III–IV/I–II) | 2.314(1.283-4.172) | 0.005 | 2.131 (1.171-3.876) | 0.013 |
| Vascular tumor invasion (macro/micro/none) | 1.911(1.214-3.008) | 0.005 | 1.806 (0.967-3.876) | 0.064 |
| Prognostic model (high risk group /low risk group) | 3.313(1.843-5.956) | 0.000 | 2.655 (1.455-4.845) | 0.001 |
